# Design and Evaluation of a Simulation for Pediatric Dentistry in Virtual Worlds

**DOI:** 10.2196/jmir.2651

**Published:** 2013-10-29

**Authors:** Lazaros Papadopoulos, Afroditi-Evaggelia Pentzou, Konstantinos Louloudiadis, Thrasyvoulos-Konstantinos Tsiatsos

**Affiliations:** ^1^ Laboratory of Medical Informatics Medical School Aristotle University of Thessaloniki Thessaloniki Greece; ^2^ Multimedia Lab (Division of Technology-Enhanced Learning) Department of Informatics of the Faculty of Sciences Aristotle University of Thessaloniki Thessaloniki Greece; ^3^ Division of Preventive Dentistry, Periodontology and Biology of Implants School of Dentistry Aristotle University of Thessaloniki Thessaloniki Greece

**Keywords:** virtual patient, virtual world, pediatric dentistry, simulation, Second Life, OpenSim, communication, tell-show-do, behavior management

## Abstract

**Background:**

Three-dimensional virtual worlds are becoming very popular among educators in the medical field. Virtual clinics and patients are already used for case study and role play in both undergraduate and continuing education levels. Dental education can also take advantage of the virtual world’s pedagogical features in order to give students the opportunity to interact with virtual patients (VPs) and practice in treatment planning.

**Objective:**

The objective of this study was to design and evaluate a virtual patient as a supplemental teaching tool for pediatric dentistry.

**Methods:**

A child VP, called Erietta, was created by utilizing the programming and building tools that online virtual worlds offer. The case is about an eight-year old girl visiting the dentist with her mother for the first time. Communication techniques such as Tell-Show-Do and parents’ interference management were the basic elements of the educational scenario on which the VP was based. An evaluation of the simulation was made by 103 dental students in their fourth year of study. Two groups were formed: an experimental group which was exposed to the simulation (n=52) and a control group which did not receive the simulation (n=51). At the end, both groups were asked to complete a knowledge questionnaire and the results were compared.

**Results:**

A statistically significant difference between the two groups was found by applying a *t* test for independent samples (*P*<.001), showing a positive learning effect from the VP. The majority of the participants evaluated the aspects of the simulation very positively while 69% (36/52) of the simulation group expressed their preference for using this module as an additional teaching tool.

**Conclusions:**

This study demonstrated that a pediatric dentistry VP built in a virtual world offers significant learning potential when used as a supplement to the traditional teaching techniques.

## Introduction

Three-dimensional (3D) virtual worlds such as “Second Life” (SL) and “OpenSimulator” (OpenSim) are becoming increasingly popular in medical education. The embedded characteristics of social networking, collaboration, constructivism, exploration, 3D building, and programming make them excellent candidates for designing teaching tools, various e-learning activities, and simulations. But what is a virtual world? According to a recent definition, a virtual world is a synchronous, persistent network of people, represented as avatars, facilitated by networked computers [1]. By “persistent”, it is implied that this virtual world, along with the changes the users make, continue to exist and evolve while the user is offline. The users of a virtual world take the form of an avatar, which is the “alter ego” of a human being that is usually represented by a 3D humanoid model. Virtual reality differs from virtual worlds. The latter term has been applied to persistent online social spaces—virtual environments that people experience as ongoing over time and that have large populations which they experience together with others as a world for social interaction [2]. Although virtual reality technology has already been applied to dental education, including operative dentistry [3], endodontics [4], orthodontics [5], and implant surgery [6], and seems to have educational benefits, virtual worlds are relatively new to this area. Virtual worlds are similar to 3D online video games, such as the popular “World of Warcraft” and “The Sims Social” on Facebook*.* According to the Entertainment Software Association, in 2012, 49% of US households owned a dedicated game console and 31% of game players were between 18-35 years old [7]. Recently, Amer et al [8] developed a video game to teach dentin bonding. Their evaluation concluded that this method of teaching was as good as a passive, non-interactive way of teaching and also dental students preferred it to a lecture. Although virtual worlds are focused on building and socializing, these facts may imply that they may also be used for creating e-learning activities that draw students’ attention.

Second Life is a virtual world, developed by Linden Lab in 2003, consisting of an online 3D environment, the avatars of the users, which are called “residents”, and the objects they create [9]. The users connect to SL from a computer using a program called “Viewer”*.* Residents can explore the world, communicate with each other, rent their own virtual home, and construct 3D objects that they can share or sell in SL’s “Marketplace”. Communication is possible via text messages, speech, and gestures. SL has its own programming language, LSL (Linden Scripting Language). In 2011, SL’s total virtual area occupied 2060 square kilometres. In order to enter the world, the user must possess an Internet connection. No offline mode is supported. The official SL viewer can be downloaded from the SL website. Third-party viewers are also available.

OpenSim is an open-source virtual world server, very similar to SL and compatible to SL’s viewer and programming language [10]. OpenSim was launched in 2007 and allows users to build their own virtual worlds and operate in online or offline modes. The latter is the main advantage of OpenSim over SL; moreover in SL, the users must own or rent a region of virtual land in order to build interactive objects. Furthermore, that land has a limitation imposed on the number of primitive geometric shapes (called “primitives” or “prims” for short). On the other hand, OpenSim is free of charge and supports any number of prims.

Until today, there have been some good efforts to create medical simulations in virtual worlds. Ohio State University’s “Medical Center” is a virtual building in SL for educational role-play and case studies [11]. “Ann Myers Medical Center” is a complete virtual hospital with detailed interactive apparatuses [12,13]. In the “Respiratory Ward” (Imperial College London), a student can meet virtual patients, listen to their breathing sounds, and make a diagnosis [14]. Auckland University’s “Medical Center” has an emergency department, an ambulance, and virtual classrooms [15]. Creutzfeldt et al [16,17] carried out a scenario-based team training of cardiopulmonary resuscitation using avatars in a virtual world. This activity seemed to be engaging and elicited positive changes in students’ subjective experiences. Wiecha et al [18] designed a postgraduate medical education program in a virtual world and concluded that the virtual world may be used in continuing medical education in order to enhance learning outcomes. Other interesting examples include “Second Health” by Imperial College London [19] and “MUVE” on “Evergreen Islands” [20]. A case which is better related to the interests of dental students is the Kentucky University virtual anatomy lab in SL, illustrating 3D maxillofacial models containing nerves and vessels [21].

Dental educators can also use virtual worlds in order to create clinical scenarios, to allow students to interact with virtual patients and practice in diagnosis and treatment planning [22,23]. There are some interesting examples of dental education applications in virtual worlds. The virtual building of Maryland Dental School in SL was created in 2009. It features a Dental Hygiene clinic, a Pediatric Dentistry room, interactive dental units, lecture rooms, and a small museum [24]. Some dental units are intended for role-play and others for self-assessment, containing case studies with images and dental history. Team-learning activities also take place on the school’s virtual island [25]. Another effort comes from Case Western Reserve University Dental School, pointing to role-play in a virtual treatment room [26].

For playing simulated scenarios with avatars, at least two users must be connected in the virtual world; one plays the patient and the other plays the doctor. Role-play is used mainly for training in interviewing techniques [27] and other nontechnical skills such as communication [28,29]. An instructor and other students can be online too, so that discussions and team activities are feasible. Difficulties arise from the fact that a second user may not always be available nor act successfully as a simulated patient. This is where Virtual Patients (VPs) apply. A VP is a specific type of computer program that simulates real-life clinical scenarios; learners emulate the roles of health care providers to obtain a history, conduct a physical exam, and make diagnostic and therapeutic decisions [30]. By utilizing VPs, students acquire the role of a doctor in a safe and controlled environment where they can develop clinical and communicative skills without the risk of disturbing or hurting people. VPs can be used for practicing interviewing [31], clinical reasoning [32], or even to facilitate the teaching of medical ethics, medical law, or medical professionalism [33]. VPs can also simulate different psychological states of a patient (angry, worried, happy, etc.) and different personalities by proper combination of programming and graphics. This is very important for the development of a student’s communication skills. Janda et al [34] found that the use of VPs improved the capability of dental students to take a health history. Another study [35] investigated the use of VPs in dental care for persons with special needs and concluded that dental students demonstrated improved communication skills and became more effective when caring for such patients.

In a recent survey [36], 63.3% of dental schools in the United States and Canada indicated that they are currently using or, at some point in the past, have used VPs in training dental students, while over 80% of the respondents, most of whom were students, seemed to enjoy the use of VPs and considered them advantageous in dental training. In a similar survey [37], 24% of the schools had developed VP case scenarios.

In contrast to adult patients, children are more difficult to simulate due to their complex or unpredictable behavior during dental practice. A dental student or an instructor can easily act as an adult patient during role-play but when it comes to pediatric dentistry, real patients are ideal for teaching behavioral techniques. Unfortunately, this may not be feasible due to practical and ethical limitations. A possible solution is to teach children to act as simulated patients in various clinical conditions [38], but considering the cost and difficulty of this procedure, it may be preferable to follow a more traditional teaching method. Interactive manikin models, multimedia software, virtual patients, or a combination of these three may be helpful in acquiring communication and behavioral skills in pediatric dentistry. Boynton et al [39] developed an Internet-based instruction tool (The Virtual Child) to simulate clinical experience in the dental treatment of a child. This study found that students who had been exposed to this simulation performed significantly better on an examination regarding knowledge of pediatric behavior management than did the control group. Kleinert et al [40] created an offline multimedia-based virtual patient model involving a dental visit for a child with Down syndrome. The study, involving 51 dental students, showed significant changes in both knowledge and perceived difficulty levels for the participants as a result of completing the module.

For our study, a virtual child patient was developed to support training of communication and behavior management in pediatric dentistry. A small 3D clinic was set in a virtual world. Our objective was to examine whether this simulation would result in increased knowledge when used as a supplementary teaching tool compared to the traditional lectures alone and to provide an evaluation of its features by the dental students.

## Methods

**Figure 1:**
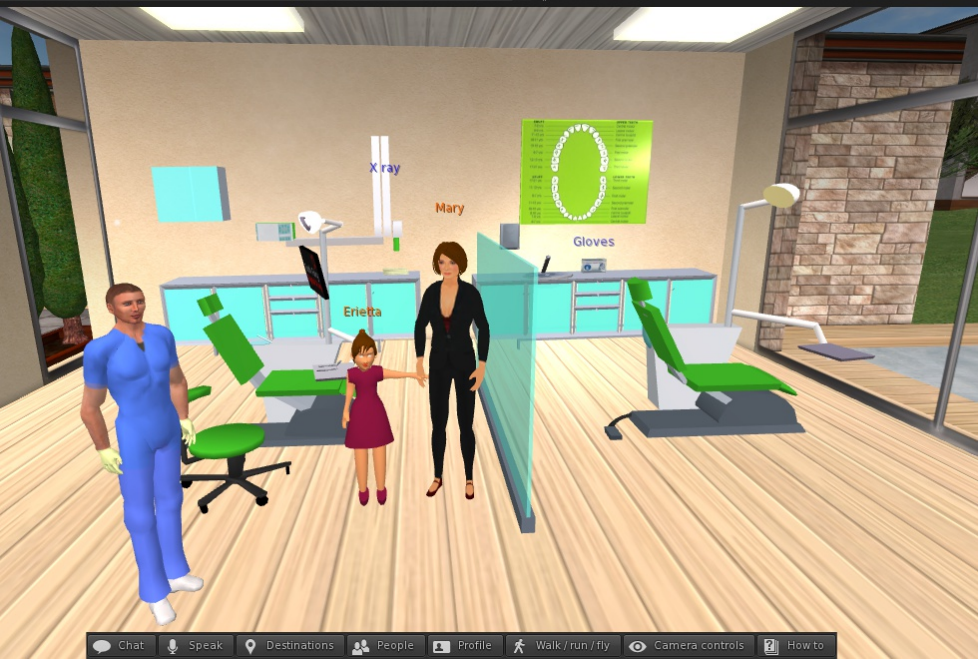
“Erietta” simulation: the treatment room.

“Erietta” is a virtual child patient designed by the authors, built in a virtual world combining 3D graphics, LSL programming, educational software principles, and communication management in pediatric dentistry. For this simulation, a small virtual dental clinic was constructed using prims and divided into two rooms. In the Tutorial Room, an interactive presentation board is placed on the wall. The user can watch slides illustrating basic techniques of communication and behavior management in pediatric dentistry. This e-learning content is based on AAPD (American Academy of Pediatric Dentistry) guidelines [41]. The Treatment Room (Figure 1) features two dental units with doctors’ seats, a digital radiograph, cabinets, water sink, and a scrubs locker. The latter contains a wearable dental uniform. A box with examination gloves is also available. A mirror and a probe are placed on the unit’s tool tray. The humanoid models of Erietta and her mother are placed standing next to a dental unit. All 3D items were designed combining “Blender” (3D software) [42] and the special build tools of SL’s viewer. The mother’s model was purchased from SL’s marketplace. Synth (synthesized) voice and other sounds such as the beeping of the x-ray were incorporated in the models to provide a more realistic simulation experience. Most of the items were animated by using special LSL functions; for example, Erietta can sit, stand, and raise her hand, the radiograph device can be unfolded, the chair can move up/down, and the light can be turned on/off.

**Figure 2:**
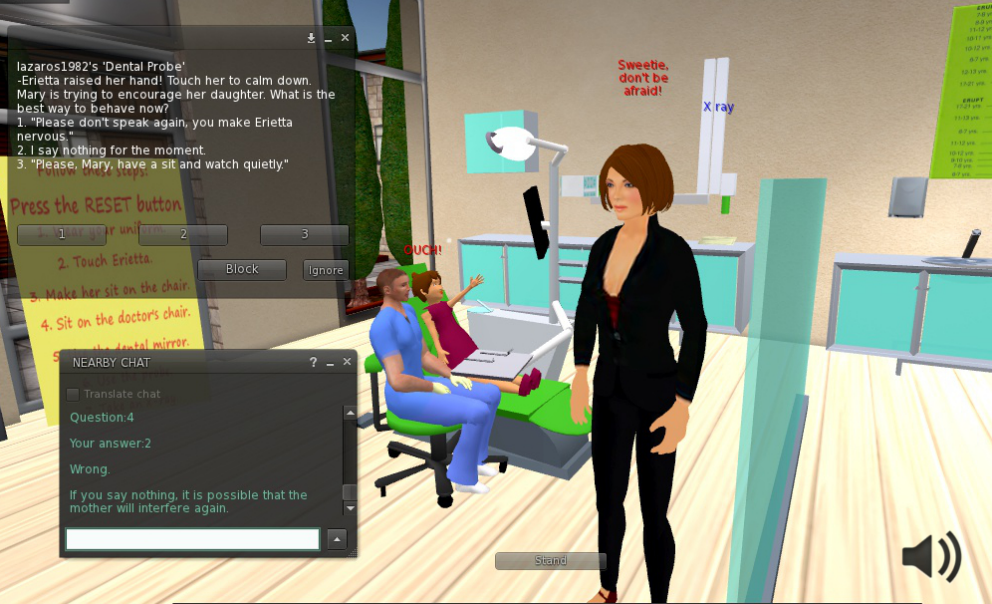
Erietta raises her hand, while her mother interferes, showing text and speech feedback.

According to our educational scenario, Erietta, an eight-year-old girl, with her mother, Mary, is visiting the dental clinic for the first time in order to have Erietta’s oral health checked. The child must first sit in the chair and then receive a simple examination. During the visit, the user will have to encounter the child’s fear and mother’s interference (Figure 2). The goal is to earn Erietta’s trust and remove any anxiety by applying basic behavior and communication techniques. The scenario consists of six parts (A: welcome, B: interference, C: examination, D: distraction, E: x-ray, and F: goodbye). Each one of them is implemented using multiple-choice questions and sound/text feedback. Conversation, Tell-Show-Do, word substitution (euphemism), distraction, and positive reinforcement are the basic methods of communication that the user is asked to apply. A sample question is shown in Table 1. Every correct answer is accompanied by a positive reaction from the child, whereas a wrong choice makes Erietta anxious and provides hints to the user. The feedback is provided in the terms of synth speech, short text appearing over the models’ heads, and detailed instructions through the chat window. Positive feedback is marked with blue fonts while negative feelings are colored red.

**Figure 3:**
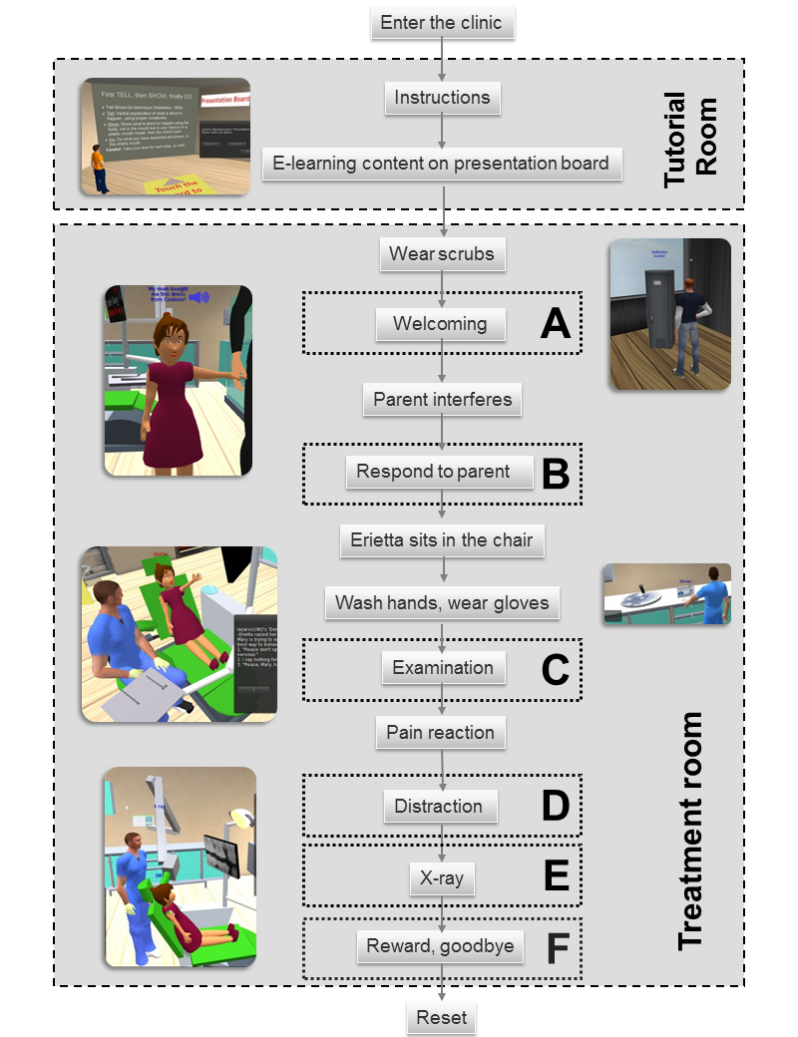
Diagram of the scenario. A: Initial communication (Questions 1-3). B: Talk to Mary (Question 4), C: Tell-Show-Do, word substitution (Questions 5-10). D: Distraction, ask Mary to be quiet (Questions 11-12). E: Tell-Show-Do, diagnosis (Questions 13-14), F: Say goodbye (Question 15).

Fifteen multiple-choice questions and their feedback were prepared and imported into Erietta using LSL and SL’s notecards. For question reading and answer finding, LSL’s API Dataserver was used. The user chooses the correct answer and proceeds to the next question. A diagram of the story is illustrated in Figure 3. There are some prerequisites in order to advance to each next step of the simulation; the user must wash hands and wear gloves prior touching the tools. A bare-hands touch event on the tools will result in a warning voice message advising the user to wear gloves. Similarly, an interaction with the radiograph device is not permitted unless Erietta is seated in the chair and the user has successfully used the mirror and probe. While an avatar is using the simulation, other users can also be online and watch, but only one avatar at a time is permitted to interact with Erietta. In this way, it is ensured that the trainee’s learning activity will be uninterrupted while a level of team-learning is provided, via watching, for the rest of the users. Two versions of the simulation were developed: one for SL and one for OpenSim. Both versions are hosted in the “Virtual Islands for Biology Education” (VIBE) [43] in their SL region and OpenSim server respectively [44,45]. For this study, the OpenSim version was preferred due to its advantageous standalone function. OpenSim server v.0.7.3 and SL viewer v.3.2.8 were installed on the PC lab’s computers in the AUTh (Aristotle University of Thessaloniki) Dental School.

A class of 103 undergraduate dental students at the Aristotle University of Thessaloniki was included in this study: 82 of the participants (79.6%, 82/103) were 25 years of age or under and the rest (20.4%, 21/103) were between the ages of 26 and 30. Of the 103 students, 53.4% (55/103) were female and 46.4% (48/103) were male. At the time of the study (2012 spring semester), the students were attending their fourth year of the curriculum and had enrolled in the Pediatric Dentistry course during the past winter semester. This course was lecture-based and included behavior guidance and communication techniques.

The class was divided into two groups. The first one, consisting of 52 students, was the experimental (simulation) group. Each student of this group was exposed to the simulation and then completed a knowledge questionnaire. The second group (control group) consisted of 51 students who answered the same questions without having used the simulation. The study was held during the Dental Informatics course and was completed in one week.

The questionnaire was designed to evaluate students’ knowledge of behavior and communication management in pediatric dentistry and consisted of seven objective multiple-choice questions. Each one of the questions focused on one of the following seven techniques: (1) welcoming, (2) tell-show-do, (3) euphemism, (4) parent management, (5) communication, (6) distraction, and (7) rewarding. A short clinical scenario describing a problem similar to the ones that the students faced in the simulation and four possible ways of reaction were offered for each question. Students were asked to select the best one. A sample question is shown in Textbox 1. Additionally, both groups answered six 3-scale questions to assess their basic knowledge of computers (duration, frequency, reason for use, 3D video games experience, and subjective level of computer skills). The students’ answers were scored individually, totaled, and entered into a spreadsheet.

The students in the simulation group filled in an evaluation questionnaire with three parts. The first part consisted of 11 Likert-scale questions to evaluate the usability and educational characteristics of the simulation and scenario. The rating was between “1” and “5” with lower scores being more positive. In the second part, students had to respond to the hypothesis of choosing whether or not to use Erietta as a teaching tool if they were instructors. The last part consisted of three open-ended questions concerning the ease of use of the virtual world, improvements and suggestions, and subjective definition of the kind of benefit gained as a result of the simulation experience (practical, cognitive, or psychological).

All the answers were scored, totaled, and entered into a spreadsheet. Then, the answers to the open-ended questions were categorized and a list of possible responses was developed for each question. All the data were exported to be analyzed with the Statistical Package for Social Sciences (SPSS).

**Table 1:** Sample question for users to answer: "After the “Tell” and “Show” steps using the probe on Erietta’s nails, what is the best way to ask Erietta open her mouth?"

Answer	Feedback in chat window	Erietta’s speech feedback
1. “Erietta, now open your mouth, so we can count your teeth!”	1. You can say that but it’s better to combine elements from the “Show” step, try again!	I don’t like it!
2. “…so, we have 10 nails on our hands! Let's see how many teeth are in your mouth! If you feel uncomfortable, raise your left hand to stop counting. Open...”	2. Correct! You applied the “Do” step for the probe.	It’s fun!
3. “Now it's time to count your teeth! If anything goes wrong, raise your left hand so I can stop counting. Open...”	3. You used the word “wrong”. This may make Erietta anxious. Try again.	Mum, I’m afraid!

**Sample question from the knowledge questionnaire.**

Dimitris is ten years old and is sitting in the chair, ready to have a primary tooth extracted. You have administered local anesthesia and explained the steps that will follow. Still, Dimitris looks frightened. How would you react?

A. You remain calm and friendly and define the length of the operation by saying: “I will count slowly to 60 and we will have finished.” You also try to distract his attention by audiovisual means (TV, music).

B. You ask his mother to help by further explaining the steps of the procedure. This will calm him down.

C. You speak strictly to Dimitris and ask him to stay calm and still, so the extraction can finish quickly. You also tune the TV on a cartoon channel.

D. You say with calm voice: “I understand you feel worried. Take your time and relax. We will start when you feel ready.”

## Results

Students of both groups completed the knowledge (objective) questionnaire. The mean total score for the simulation group was 5.40 (SD 1.40). The mean total score for the control group was 3.61 (SD 1.44). The maximum possible score was 7. A *t* test for independent samples showed a statistically significant difference between the groups, indicating a gain in knowledge for the students who were exposed to the simulation: *t*_101_=6.40, *P*<.001 (Table 2). The simulation group had more correct responses than the control group in all seven questions. The greatest difference was noticed in the first question, in which 45 students of the simulation group (87%, 45/52) marked the correct choice, in contrast to only 20 students (39%, 20/51) of the control group. Table 3 shows the scores of the two groups for every question.

In order to assess the potential effect of previous experience with computers and/or 3D games on examination results, data were analyzed to reveal any differences between the two groups. Chi-square analysis of the above-mentioned features found no significant differences between the two groups. However, some interesting results were extracted. In total, 92 of the participants (89.3%, 92/103) had been using computers for more than four years and 51 students (52%, 51/98) subjectively defined their computer skills as “moderate”. Also, 90 of the students (91%, 90/98) were using computers for fun and education and 59 students (60%, 59/98) had played 3D games, of whom, 29 (49%, 29/59) had been playing 3D games for over four years (Table 4).

All 52 students of the simulation group completed the evaluation questionnaire. Students agreed that the simulation, overall, was very comprehensible (rating 1.52 on the scale), very easy to use (1.71), very educative (1.85), very interesting (1.99), very pedagogical (1.92), and original (1.98). Regarding the individual characteristics of the scenario, students rated it as very comprehensible (1.58), very pedagogical (1.88), very educative (1.89), very well-aimed (1.92), and very interesting (2.06). Mean ratings and standard deviations for the Likert-scale questions are illustrated in Tables 5 and 6. Further, 36 students from the simulation group (69%, 36/52) declared that they would use the “Erietta” simulation as a teaching tool if they were instructors. Interestingly, a cumulative 92.3% answered “Yes” or “Maybe” to that question (Table 7).

In the first open-ended question, 50 students (96%, 50/52) responded that the virtual world was very easy to use. In the suggested improvements question, 24 students (46%, 24/52) had no changes to suggest, 5 students (10%, 5/52) would like a variety of selectable scenarios, 3 students (6%, 3/52) asked for better graphics, and 2 students (4%, 2/52) suggested that the dialogs and menus be in Greek (native language). In the last question, 19 students (37%, 19/52) believed that the simulation helped them in all three levels: practical, psychological, and cognitive; 12 students (23%, 12/52) answered “practical” only, 6 students (12%, 6/52) answered “cognitive” only, and 5 students (10%, 5/52) answered “psychological” only (Table 8).

**Table 2:** Knowledge test scores of the two groups.

Knowledge questionnaire	Simulation (n=52)			Control (n=51)		
	Mean	SD	Std Error Mean	Mean	SD	Std Error Mean
Multiple-choice questions score	5.40	1.40	0.19	3.61	1.44	0.20

**Table 3:** Correct answers count per question in knowledge questionnaire.

Knowledge questions	Simulation (n=52)		Control (n=51)	
	Frequency	Percent	Frequency	Percent
Q1. Greeting/welcoming the child	45	87	20	39
Q2. Tell-Show-Do	30	58	18	35
Q3. Word substitution	30	58	15	29
Q4. Parent’s interference	47	90	36	71
Q5. Communication	45	87	30	59
Q6. Distraction	47	90	35	69
Q7. Rewarding	37	55	30	45

**Table 4:** Computer skills profile of the participants (N=103).

Experience in computer usage		Simulation (n=52)		Control (n=51)		Percent of total count
		Frequency	Percent	Frequency	Percent	
**Years of computer usage**						
	0-1	0	0.0	0	0.0	0.0
	2-4	6	11.5	5	9.8	10.7
	>4	46	88.5	46	90.2	89.3
**Subjective level of computer skills**						
	Extremely good	16	32.7	17	34.7	33.7
	Moderate	24	49.0	27	55.1	52.0
	Basic	9	18.4	5	10.2	14.3
**Primary reason for computer usage**						
	Education	1	2.0	0	0.0	1.0
	Fun	3	6.1	4	8.2	7.1
	Both	45	91.8	45	91.8	91.8
**Have played 3D games**						
	Yes	30	61.2	29	59.2	60.2
	No	19	38.8	20	40.8	39.8
**Years of playing 3D games (If “Yes” was answered to the previous question)**						
	<2	9	30.0	8	27.6	28.8
	2-4	10	33.3	3	10.3	22.0
	>4	11	36.7	18	62.1	49.2

**Table 5:** Students’ evaluation of “Erietta” simulation overall, on a scale of 1 (extremely) to 5 (not at all) (n=52).

Characteristic	Mean	SD
Comprehensible	1.52	0.87
Easy to use	1.71	1.02
Educative	1.85	1.14
Interesting	1.99	1.11
Pedagogical	1.92	1.04
Original	1.98	1.22

**Table 6:** Students’ evaluation of the simulation scenario on a scale of 1 (extremely) to 5 (not at all) (n=52).

Characteristic	Mean	SD
Comprehensible	1.58	0.94
Educative	1.89	1.02
Aimed	1.92	0.89
Interesting	2.06	1.02
Pedagogical	1.88	1.15

**Table 7:** Students’ answers to the question: “Would you use this simulation as a teaching tool if you were an instructor?” (n=52).

Answer	Frequency	Cumulative percent
Yes	36	69.2
Maybe	12	92.3
No	4	100

**Table 8:** Students’ answers to open-ended questions (n=52).

Categorized answers		Frequency	Percent
**Was the virtual world easy to use? Please justify your answer.**			
	Very easy	50	96
	Difficult if you don’t have previous computer knowledge	2	4
**What changes or improvements would you suggest?**			
	More scenarios to select from	5	10
	More comprehensive dialogs	1	2
	Greek menus/dialogs	2	4
	No changes	24	46
	Better graphics	3	6
	Quicker and easier in use	1	2
	Other	8	15
	Did not respond	8	15
**Do you believe that the simulation helped you in a cognitive, practical, or psychological way? Please justify your answer.**			
	All three ways	19	37
	Practical only	12	23
	Practical and psychological	5	10
	Psychological only	5	10
	Cognitive only	6	12
	Cognitive and practical	3	6
	Other	1	2
	Did not respond	1	2

## Discussion

### Principal Findings

The objective of our study was to measure the efficacy of a child VP in a virtual world, as a supplementary teaching tool for pediatric dentistry. This simulation aimed to support training in communication and behavior techniques. Previous research has shown that VPs can be used for practicing on patient interviewing, communication, and clinical reasoning [30-34]. For this study, a combination of VPs and virtual worlds was attempted. Virtual worlds offer an online programming and 3D building environment in which users can meet and socialize. Previous efforts on creating children VPs, such as the online text-based “Virtual Child” [39] and Kleinert’s interactive video-based module [40], resulted in knowledge gain and were evaluated positively by the students. Similarly, our simulation group achieved significantly better results than the control group did in the knowledge questionnaire. This indicates that the simulation acted as a supplement to the lectures of the pediatric dentistry course. More than one-third of the students admitted that this experience helped them in three levels: practical, cognitive, and psychological, while a significant number of answers concentrated in practical level only. This is a very interesting result, taking into account the lack of adequate practical training in communication techniques at the University. The students rated the educational aspects of the simulation highly, such as the comprehensiveness, pedagogical value, ease of use, and originality. The scenario was also accepted very well, although it was simple and linear. Almost half of the participants had no improvements to suggest, some students asked for a variety of selectable scenarios, and a few others suggested better graphics. These changes are easy to implement due to the modular nature of the simulation: Erietta is a VP made of simple 3D graphics, LSL scripts, and notecards. The virtual world offers the capability for expansions and add-ons to be designed online or offline and then uploaded, or purchased by the Marketplace and then programmed to operate at the designer’s will. New questions can also be added, as notecards.

Although no difference was found between the two groups regarding to their skills on computers and 3D games, most of the students were found to have been using computers for more than four years. More than half of the participants had played 3D games. It must be noted that the current generation of dental students differs a lot from older ones, in terms of technology knowledge. Today’s students could be characterized as aborigines of a “computer era”; most of them have been using computers and/or playing video-games since their childhood. This fact indicates that technologies such as VPs, virtual worlds, serious games, social networks, and the upcoming Web 3.0 are almost a requisite in the education of new healthcare providers. In our study, the majority of the students agreed that they would use Erietta as a teaching tool if they were instructors. This finding proves that such tools are welcome in dental education and will be happily accepted by the students. Being inside a virtual environment as an avatar along with your colleagues and exploring interactive 3D items from your home PC without the need of supervision may have great e-learning potential and also reduce faculty working time. It must be stressed that these new methods of creating educational material are intended to support the traditional learning methods, not to replace them; in this study, a simulation was utilized as a teaching supplement, not a replacement.

### Limitations

The evaluation did not include measures of changes in students’ skills on behavior management. Ideally, an assessment of communication skills with a child in a real dental operatory should have been made. Although this would require the presence of children, it is planned as a future work, while a new version of the Erietta simulation is currently under development.

All the participants had already completed the Pediatric Dentistry course, so a homogeneous status of knowledge was hypothesized at the beginning of the evaluation. For this reason, the knowledge assessment was not measured at baseline and hence it is difficult to have a clear picture of the significance of the difference between the two groups.

Virtual worlds require computers with good graphics cards and an Internet connection. Although creating simulations with relatively simple graphics such as Erietta is easy, detailed models demand more effort and special software. Programming experience is also required in order to write a script in LSL. For this study, a linear scenario consisting of six steps was written. No branches or alternative ways of achieving the goal were designed because of time limitations. Also, our objective was to examine the efficiency of a pediatric VP in virtual worlds, by utilizing a less sophisticated scenario. Though, as a future improvement, we are planning to expand the current simulation by adding more steps and branches to each selection and creating a pool of stories with various difficulty for the user to select.

### Conclusions

This project has indicated that a simulation based on a virtual patient, built in a virtual world, may improve student knowledge in communication management for pediatric dentistry when used as a supplementary teaching tool.

## Abbreviations

AAPD: American Academy of Pediatric Dentistry
OpenSim: Open Simulator
SL: Second Life
VP: virtual patient
