# Metadata Correction: Design and Evaluation of a Simulation for Pediatric Dentistry in Virtual Worlds

**DOI:** 10.2196/jmir.3134

**Published:** 2013-11-26

**Authors:** Lazaros Papadopoulos, Afroditi-Evaggelia Pentzou, Konstantinos Louloudiadis, Thrasyvoulos-Konstantinos Tsiatsos

**Affiliations:** ^1^ Laboratory of Medical Informatics Medical School Aristotle University of Thessaloniki Thessaloniki Greece; ^2^ Multimedia Lab (Division of Technology-Enhanced Learning) Department of Informatics of the Faculty of Sciences Aristotle University of Thessaloniki Thessaloniki Greece; ^3^ Division of Preventive Dentistry, Periodontology and Biology of Implants School of Dentistry Aristotle University of Thessaloniki Thessaloniki Greece

“Design and Evaluation of a Simulation for Pediatric Dentistry in Virtual Worlds” (J Med Internet Res 2013;15(10):e240) was published on Oct 29th in issue 10 (with correct citation information in the table of contents) but the article itself originally displayed incorrect “please cite as” citation information, displaying issue 11 instead of issue 10.  This error was corrected in the online version of the paper on the JMIR website on November 26, 2013, together with publishing this correction notice. As a result, the URL for this paper has changed from http://www.jmir.org/2013/11/e240/ to http://www.jmir.org/2013/10/e240/. No content changes were made.

A correction notice has been sent to PubMed. This was done before submission to Pubmed Central and other full-text repositories.

